# A revised view on the evolution of glutamine synthetase isoenzymes in plants

**DOI:** 10.1111/tpj.15712

**Published:** 2022-03-09

**Authors:** José Miguel Valderrama‐Martín, Francisco Ortigosa, Concepción Ávila, Francisco M. Cánovas, Bertrand Hirel, Francisco R. Cantón, Rafael A. Cañas

**Affiliations:** ^1^ Grupo de Biología Molecular y Biotecnología, Departamento de Biología Molecular y Bioquímica Universidad de Málaga, Campus Universitario de Teatinos 29071 Málaga Spain; ^2^ Institut National de Recherche pour l'Agriculture, l'Alimentation et l'Environnement (INRAE), Centre de Versailles‐Grignon RD 10 78026 Versailles Cedex France; ^3^ Integrative Molecular Biology Lab Universidad de Málaga, Campus Universitario de Teatinos 29071 Málaga Spain

**Keywords:** adaptation, glutamine synthetase, new gene classification, nitrogen metabolism, phylogeny, plant evolution

## Abstract

Glutamine synthetase (GS) is a key enzyme responsible for the incorporation of inorganic nitrogen in the form of ammonium into the amino acid glutamine. In plants, two groups of functional GS enzymes are found: eubacterial GSIIb (GLN2) and eukaryotic GSIIe (GLN1/GS). Only *GLN1/GS* genes are found in vascular plants, which suggests that they are involved in the final adaptation of plants to terrestrial life. The present phylogenetic study reclassifies the different *GS* genes of seed plants into three clusters: *GS1a*, *GS1b* and *GS2*. The presence of genes encoding GS2 has been expanded to Cycadopsida gymnosperms, which suggests the origin of this gene in a common ancestor of Cycadopsida, Ginkgoopsida and angiosperms. *GS1a* genes have been identified in all gymnosperms, basal angiosperms and some Magnoliidae species. Previous studies in conifers and the gene expression profiles obtained in ginkgo and magnolia in the present work could explain the absence of *GS1a* in more recent angiosperm species (e.g. monocots and eudicots) as a result of the redundant roles of GS1a and GS2 in photosynthetic cells. Altogether, the results provide a better understanding of the evolution of plant GS isoenzymes and their physiological roles, which is valuable for improving crop nitrogen use efficiency and productivity. This new view of GS evolution in plants, including a new cytosolic GS group (GS1a), has important functional implications in the context of plant metabolism adaptation to global changes.

## Significance Statement

We provide a new view of glutamine synthetase evolution in plants, including a new cytosolic GS group (GS1a), with important functional implications in the context of plant metabolism adaptation to global changes.

## INTRODUCTION

Glutamine synthetase (GS, EC 6.3.1.2) catalyzes the incorporation of ammonium into glutamate using ATP to produce glutamine while releasing Pi and ADP (Heldt and Piechulla, 2011). GS is an enzyme of major importance, as it represents the main, if not the only, mechanism incorporating inorganic nitrogen (N) into organic molecules in virtually all living organisms (Shatters and Kahn, 1989). It has been suggested that the genes encoding GS are not only some of the oldest genes in evolutionary history (Kumada et al., 1993) but also represent an excellent ‘molecular clock’ that can be used to perform phylogenetic studies (Pesole et al., 1991).

Three GS superfamilies have been identified, namely GSI, GSII and GSIII, with the corresponding proteins characterized by different molecular masses, different numbers of subunits and their occurrence in the three different domains of life (Archaea, Bacteria and Eukarya) (Ghoshroy et al., 2010). The GSI superfamily was first found in prokaryotes, although its presence in mammals and plants has also been reported (Mathis et al., 2000 **;** Nogueira et al., 2005 **;** Kumar et al., 2017). The GSII superfamily was described as a group characteristic of Eukarya and some Bacteria, such as Proteobacteria and Actinobacteria (James et al., 2018). However, the nucleotide sequences deposited in public databases indicate that this GS superfamily is also present in Euryarchaeota, a phylum of the Archaea domain. Finally, the GSIII superfamily is characteristic of bacteria, including cyanobacteria (James et al., 2018), and some eukaryotes, such as diatoms and other heterokonts, suggesting that GSIII is present in the nuclear genome of early eukaryotes (Robertson and Tartar, 2006). The hypothesis that these three gene superfamilies appeared prior to the divergence of eukaryotes and prokaryotes has been proposed in several studies (Robertson and Tartar, 2006).

In plants, glutamine synthesis is catalyzed by enzymatic proteins belonging to the GSII superfamily. Two main groups of GSII have been shown to occur in the Viridiplantae group, one of eukaryotic origin, GSII (GSIIe), and the other of eubacterial origin, GSII (GSIIb). *GSIIb* genes are the result of horizontal gene transfer (HGT) following the divergence of prokaryotes and eukaryotes, which in turn represent a sister group of γ‐proteobacteria *GSII* (Tateno, 1994; Ghoshroy et al., 2010). As N is one of the main limiting nutrients for plant growth and development, the functions and characteristics of GS have been studied extensively in a large number of vascular plant species, and particularly in crops (Plett et al., 2017; Mondal et al., 2021). It is generally indicated that angiosperms contain two groups of nuclear genes encoding GSIIe, represented by cytosolic GS (GS1) and plastidic GS (GS2), each playing distinct physiological roles (Ghoshroy et al., 2010; Hirel and Krapp, 2021). GS2 is generally encoded by a single gene, whereas GS1 is encoded by a small multigene family (Cánovas et al., 2007; James et al., 2018). Phylogenetic analyses suggest that *GS2* probably evolved from *GS1* gene duplication (Biesiadka and Legocki, 1997) that diverged from a common ancestor 300 million years ago (Mya). Therefore, this gene duplication probably occurred before the divergence of monocotyledons and dicotyledons (Bernard and Habash, 2009). Interestingly, the gene encoding GS2 is present in the gymnosperm *Ginkgo biloba* (García‐Gutiérrez et al., 1998; Guan et al., 2016). This gene is absent in all the other gymnosperms examined thus far, including conifers (Coniferopsida) and the Gnetales (Gnetopsida), in which the gene encoding GS2 has not been found in their genomes (Birol et al., 2013; Nystedt et al., 2013; Neale et al., 2014; Zimin et al., 2014; Stevens et al., 2016; Neale et al., 2017; Wan et al., 2018; Kuzmin et al., 2019; Mosca et al., 2019; Scott et al., 2020). Furthermore, this gene also seems to be absent in cycads (Cycadopsida), as the GS2 protein was not detected in Western blot analyses (Miyazawa et al., 2018).

In both angiosperm and gymnosperm plants, the synthesis and relative activity of the different GS isoforms are regulated in a species‐specific manner, but also according to plant developmental stages, tissue, N nutritional status and environmental conditions (Cánovas et al., 2007; Bernard and Habash, 2009; Mondal et al., 2021). Consequently, each GS isoform plays a different role during N assimilation and N remobilization throughout the life cycle of a plant (Thomsen et al., 2014; Hirel and Krapp, 2021). GS2 predominates in photosynthetic tissues, such as leaf mesophyll cells, in order to assimilate the ammonium generated from nitrate reduction and released during photorespiration (Blackwell et al., 1987; Wallsgrove et al., 1987; Tegeder and Masclaux‐Daubresse, 2017). In contrast, GS1 is present in almost all plant organs and tissues (Lea and Miflin, 2018). Cytosolic GS isoforms are mostly involved in primary N assimilation in roots and N remobilization and translocation in shoots (Thomsen et al., 2014). As such, it has been shown that they play a key role during plant growth and development, notably for biomass and storage organ production (Xu et al., 2012; Krapp, 2015; Havé et al., 2017; Amiour et al., 2021). In conifers, as a result of the absence of GS2, studies have focused on GS1a and GS1b, which are each encoded by a single gene. These two cytosolic isoforms of GS also exhibit distinct molecular and kinetic properties (Ávila‐Sáez et al., 2000; de la Torre et al., 2002). GS1a has been proposed to fulfill the same function as GS2 in angiosperms because of its close relationship with chloroplast development and the presence of ammonium arising from photorespiration. This hypothesis was also supported by the fact that the gene encoding GS1a is expressed in photosynthetic organs, notably in chlorophyllous parenchyma cells (Ávila et al., 2001), and that its expression is also upregulated in the presence of light (Cantón et al., 1999; Gómez‐Maldonado et al., 2004). Moreover, GS1b is phylogenetically and functionally more closely related to the cytosolic isoforms of GS in angiosperms than to those of GS1a in conifers (Ávila‐Sáez et al., 2000; Cánovas et al., 2007).

In this work, the increasing number of plant genome sequences made available in public databases were gathered to perform a deep phylogenetic analysis of the GSII family. The present study includes representative GS sequences from the entire plant evolutionary spectra, including those from monocot and dicot angiosperms and a number of model species that were representative of other taxa. This new phylogenetic study allowed us to propose a revised classification and nomenclature for the different GS isoforms in seed plants. In addition, *GS* gene expression experiments were conducted in *G. biloba*, *Magnolia grandiflora* and *Pinus pinaster* in order to strengthen the results obtained in the GS1a phylogeny.

## RESULTS

**Figure 1:**
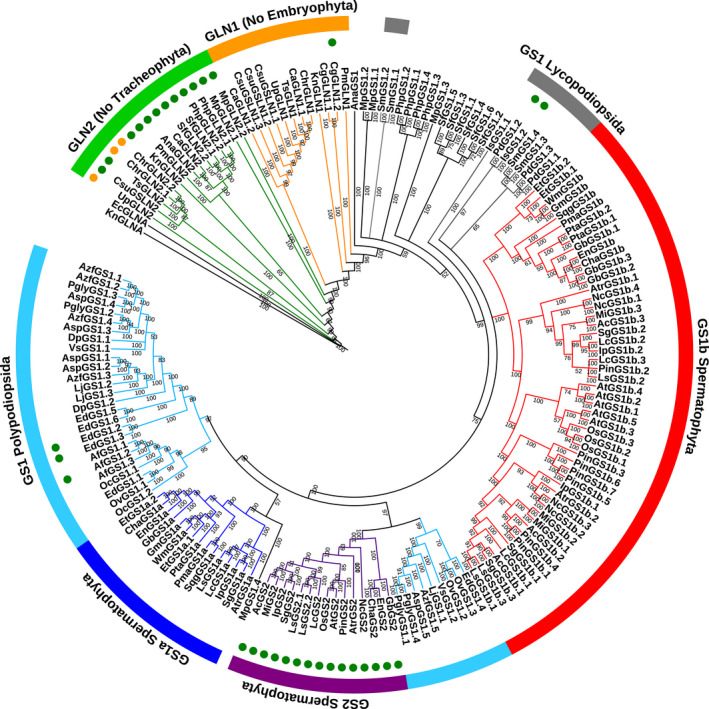
Phylogenetic tree of plant glutamine synthetase (GS) nucleotide sequences obtained following a Bayesian analysis. The first two letters of the sequence names correspond to the genera and species listed in Table S1. Green circles highlight the sequences exhibiting a predicted plastidic localization. Orange circles highlight the sequences exhibiting a predicted mitochondrial localization. Branch lengths are not presented. [Colour figure can be viewed at wileyonlinelibrary.com]

**Figure 2:**
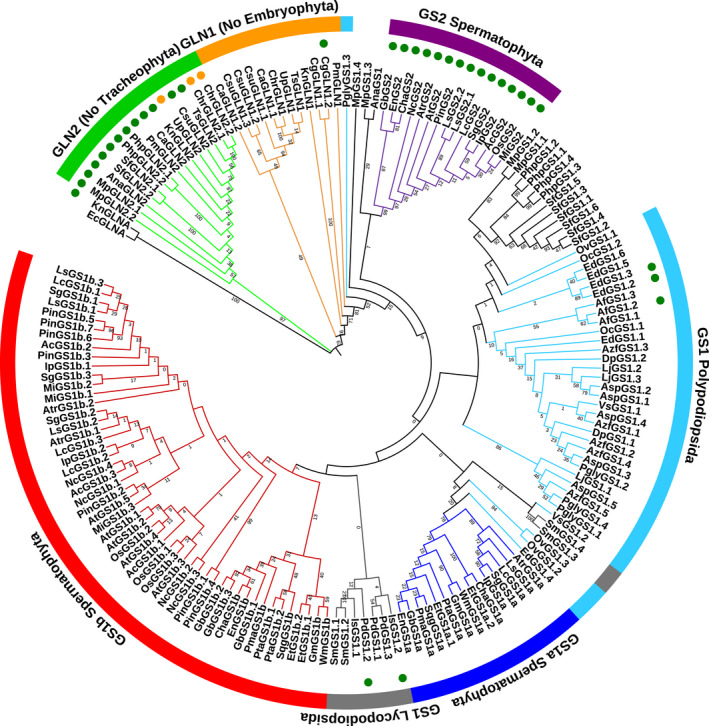
Protein phylogenetic tree of plant glutamine synthetase (GS) protein sequences following a maximum‐likelihood analysis. The first two letters of the sequence names correspond to the genera and species listed in Table S1. Green circles highlight the sequences with a predicted chloroplastic localization. Orange circles highlight the sequences with a predicted mitochondrial localization. Branch lengths are not presented. [Colour figure can be viewed at wileyonlinelibrary.com]

A total of 168 nucleotide sequences from the coding DNA sequences (CDSs) and the corresponding protein sequences of the genes encoding GSII from 45 different Viridiplantae species were retrieved from different public databases or assembled using next‐generation sequencing (NGS) data from the Sequence Read Archive (SRA) database (Table S1). Additionally, *Escherichia coli glnA* (GSI) was used as an external group. The sequences and species analyzed cover the evolutionary history of Viridiplantae and included members of the main Viridiplantae clades, unless the sequences were not available in the public databases. These sequences were used to perform phylogenetic analyses to assess GSII evolution in Viridiplantae. The final names of the sequences were assigned depending on phylogenetic analyses (Figures 1, 2; Table S1). The GSIIb sequences were named GLN2 following the nomenclature of *GLN2*, the gene encoding GS in *Chlamydomonas reinhardtii*. The GSIIe group, which corresponds to species older than the Embryophyta, was named GLN1. The sequences from Embryophyta species were named GS1, except those included in the group of the Spermatophyta GS2 sequences.

The phylogenetic studies were conducted with nucleotide sequences using Bayesian analyses (Figure 1; Table S1). For the protein sequences, a maximum‐likelihood approach was used (Figure 2; Table S2). The results were similar for the main GS groups whether the nucleotide or the protein sequences were analyzed. The *KnGLNA* sequence of *Klebsormidium nitens*, a charophyte green algae, was the most divergent GS close to the outer sequence *EcGLNA* (GSI) of *E. coli* (Figures S1 and S2). Both sequences were very distant from the other plant *GS* genes (mean length 2.0756), with a node/branch probability of 1 in the Bayesian analysis.

The first cluster contained all GLN2 (GSIIb) sequences. Notably, no GLN2 sequence was identified in vascular plants (Tracheophyta). In the protein sequence analyses, we observed that the GLN2 cluster shared a common origin. In addition, different subgroups for GLN2 were identified in the nucleotide sequence clustering analyses. These GLN2 subgroups were distant from the other plant GS sequences (GSIIe; mean length 0.7142) (Figures S1 and S2). For both the GLN2 nucleotide and protein sequences, the node/branch probability/bootstrap was high (>0.65). In all GLN2 sequences, we found a predicted localization in the chloroplast using targetp, except for UpGLN2, ChrGLN2.1 and ChrGLN2.2, for which mitochondrial localization (Figures 1 and 2; Table S1).

The most ancient GSIIe plant sequences were named GLN1, and in both analyses, they were distributed in a main group containing four non‐clustered sequences, including *KnGLN1* (Klebsormidiophyceae class), *CgGLN1.1* and *CgGLN1.2* (Coleochaetophyceae class), and *PmGLN1* (Zygnemophyceae class). These four sequences were in an intermediary position between the main GLN1 cluster and the other GS sequences from land plants. A predicted chloroplast localization was only found for CgGLN1.2 (Figures 1 and 2).

Three clusters were identified in seed plants (Spermatophyta), including plastidic GS2 and gymnosperm GS1a‐like and GS1b‐like sequences (Figures 1 and 2). Interestingly, within the GS2 group, three sequences from gymnosperms were identified (*GbGS2*, *ChaGS2* and *EnGS2*). They corresponded to a ginkgo sequence and two Cycadopsida sequences. The GS1a‐like group contained known GS1a sequences from gymnosperms and GS1 from basal angiosperms and from some Magnoliidae, except Ranunculales, Proteales, Liliopsida and Eudycotyledon species. However, an ortholog of the *GS1a* gene was not identified in the genome of *Piper nigrum*, a Magnoliidae species from the Piperales order (Hu et al., 2019). Finally, the GS1b‐like cluster contained GS1b found in gymnosperms and the GS1 enzymes previously characterized in angiosperms.

The phylogeny of GS from Anthocerotophyta, Bryophyta, Lycopodiopsida, Marchantiophyta and Polypodiopsida was more complex than that of Spermatophyta, especially when the protein sequences were analyzed (Figures 1 and 2). However, with the cognate gene sequences, Anthocerotophyta, Bryophyta, Lycopodiopsida and Marchantiophyta were in a basal position compared with the other Embryophyta species. Such a distribution corresponded to the expected evolutionary relationships between plant species, except for the outlier sequence *MpGS1.4* that was found in the GS1a‐like cluster (Figure 1). Fern (Polypodiopsida) *GS* genes were grouped into two clusters. The first one contained most of the nucleotide sequences linked to the GS1a‐like sequences, and the second was grouped with GS2 and was composed of *AspGS1.5*, *AzfGS1.5*, *EdGS1.4*, *LjGS1.1*, *OvGS1.2*, *OvGS1.3*, *PglyGS1.1*, *PglyGS1.4* and *VsGS1.1*. For these two clusters, the mean probabilities were very high (0.9 and 0.97, respectively) when Bayesian analysis was used (Figures 1 and S1).

In contrast, the phylogenetic relationships with the protein sequences were unclear because of a different cluster distribution and the occurrence of outlier sequences such as PglyGS1.3 and MpGS1.4 (Figure 2). Most of the Marchantiophyta and Bryophyta GS enzymes were grouped with most of the Polypodiopsida sequences, although MpGS1.3 and AnaGS1 clustered with the GS2 from Spermatophyta. This group of GS proteins was closer to that of the Spermatophyta GS1 compared with GS2, even though the node/branch bootstraps were very low (<1). The Lycopodiopsida GS enzymes were grouped together with GS1b‐like protein sequences even though the node/branch bootstrap was also very low (<1). Nevertheless, two sequences (SmGS1.3 and SmGS1.4) were grouped with the Spermatophyta GS1a cluster together with four Polypodiopsida sequences (EdGS1.4, OvGS1.2, OvGS1.3 and VsGS1.2) (Figure 2).

As expected, for all members of GS2, the presence of a signal peptide that allows the targeting of the protein to the chloroplast was predicted (Figures 1 and 2; Table S1). Only five of the remaining Embryophyta proteins were predicted to be localized in the chloroplast, including IsGS1.2 and PdGS1.2 from Lycopodiopsida species and EdGS1.2, EdGS1.3 and AfGS1.2 from Polypodiopsida species. Moreover, one could observe that these five GS enzymes did not belong to the GS2 cluster (Figures 1, 2; Table S1).

### Spermatophyta *GS* gene expression

To further decipher the role of GS1a in ginkgo and in angiosperms, the level of expression of different *GS* genes was quantified in *G. biloba*, *M. grandiflora* and *P. pinaster*. Maritime pine (*P. pinaster*) was included in the study because the role and gene expression pattern of *GS1a* is well established in this gymnosperm, characterized by the absence of a gene encoding GS2 (Cánovas et al., 2007). The gymnosperm *G. biloba* and the angiosperm *M. grandiflora* were also studied because they possess *GS* genes corresponding to the three main Spermatophyta GS groups (GS1a, GS1b and GS2).

**Figure 3:**
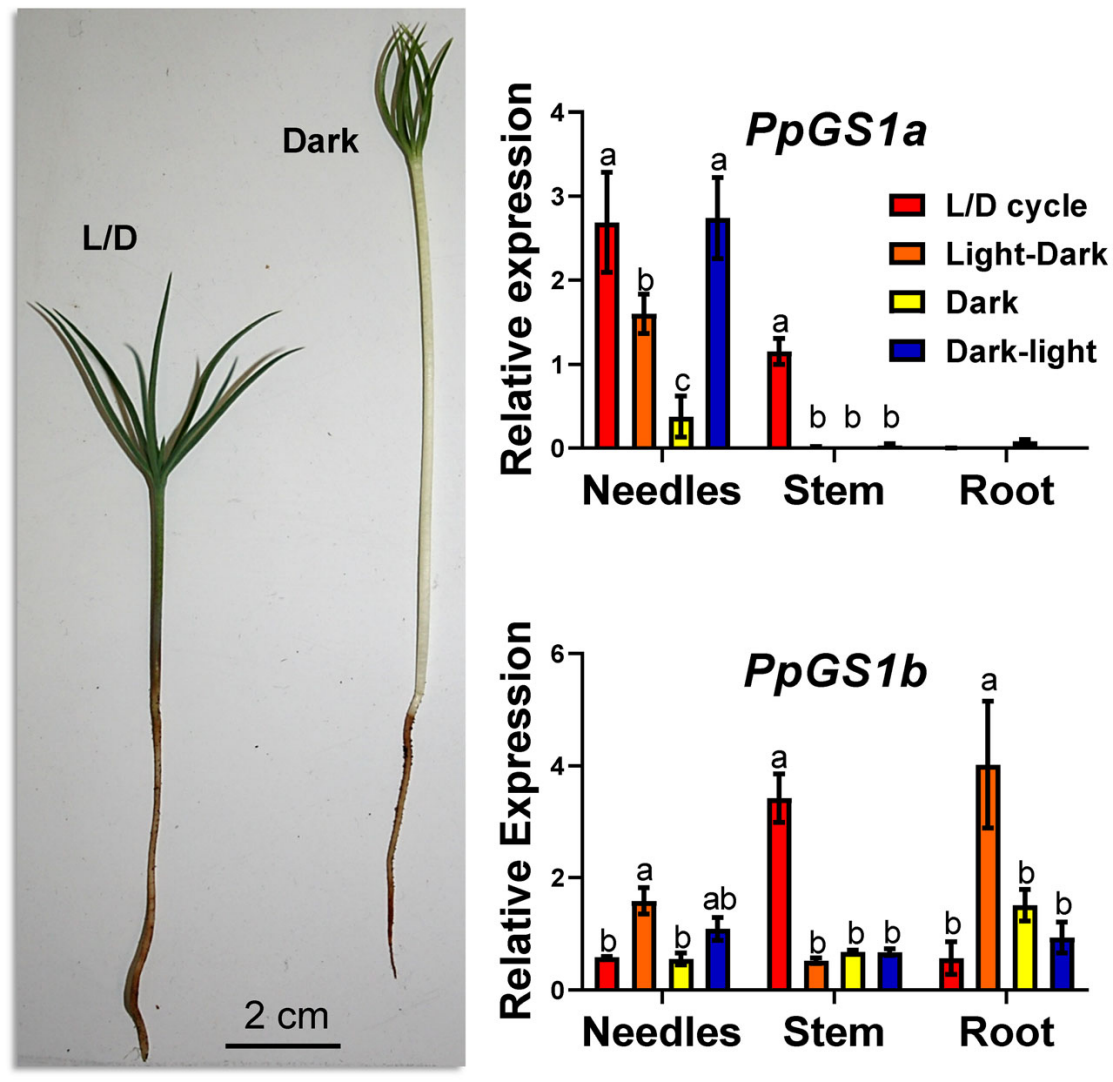
*PpGS1a* and *PpGS1b* gene expression in *Pinus pinaster* seedlings grown under different light regimes. L/D cycle (16‐h light /8‐h dark photoperiod, red bars), transition from light photoperiod to continuous darkness (orange bars), continuous darkness (yellow bars) and transition from complete darkness to light photoperiod (blue bars). Significant differences were determined using two‐way analysis of variance (ANOVA) that compares the mean for each condition with the mean of the other condition in the same organ. Letters above the columns indicate significant differences on a Tukey’s *post hoc* test (*P* < 0.05). [Colour figure can be viewed at wileyonlinelibrary.com]

In maritime pine seedlings, the profiles of *PpGS1a* and *PpGS1b* gene expression were analyzed under different light/dark regimes (Figure 3): germination with a light/dark (L/D) cycle (16 h of light/8 h of darkness), continuous darkness and two opposite nychthemeral regimes (from light to dark and from dark to light). *PpGS1a* was mainly expressed in the needles, irrespective of the light/dark regime, and in the stem only during the L/D cycle. In roots, the *PpGS1a* expression level was at the limit of detection under the four different light/dark conditions. In the needles, *PpGS1a* reached the highest level of expression in the dark–light transition, even though it was slightly lower under the L/D cycle. Compared with these two conditions, the *PpGS1a* expression level in the needles was at least five times lower when the plants were placed under continuous darkness and approximately two times lower following a light–dark transition. In contrast, *PpGS1b* was expressed in all three organs. In the needles and in the roots, the expression level of *PpGS1b* was significantly higher only during the light–dark transition. In the stem, the expression level of *PpGS1b* was highest when the seedlings were grown under the L/D cycle.

**Figure 4:**
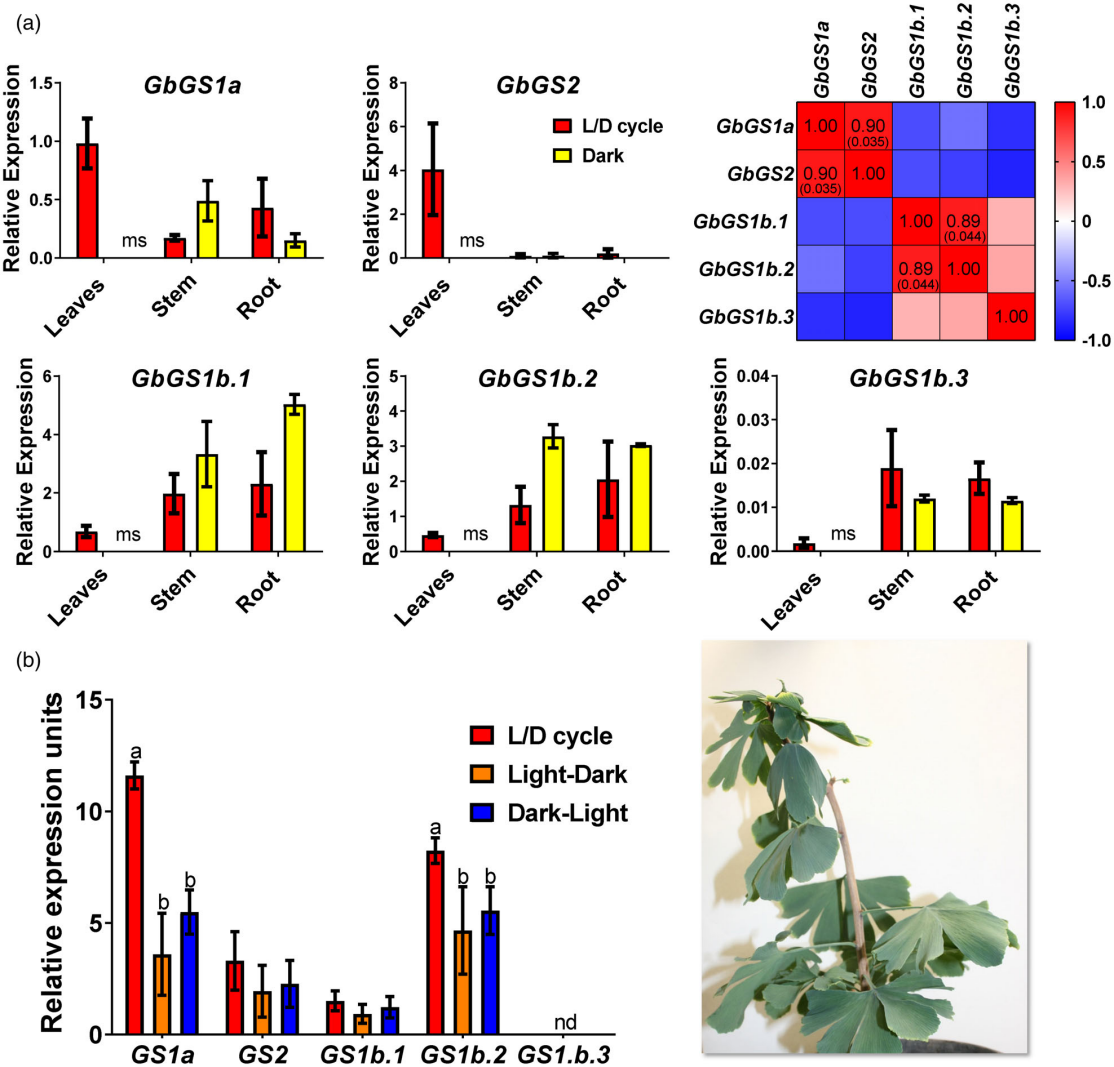
Glutamine synthetase (GS) gene expression in *Ginkgo biloba* seedlings grown under different light regimes. (a) One‐month‐old seedlings grown under the L/D cycle (16‐h light/8‐h dark photoperiod, red bars) and under continuous darkness (yellow bars). A Pearson correlation test was applied to the expression level of the different *GS* genes to quantify their relationship, indicated in the red squares. The significant *P* values (<0.05) for the Pearson coefficient are indicated in brackets. (b) One‐year‐old seedlings grown under the L/D cycle (16‐h light/8‐h dark photoperiod, red bars), under transition from the light photoperiod to continuous darkness (orange bars) and under transition from complete darkness to light photoperiod (blue bars). Significant differences were determined using a two‐way analysis of variance (anova) to compare the mean for each growth condition with the mean of the other conditions in the same organ. Letters above the columns indicate significant differences based on a Tukey’s *post hoc* test (*P* < 0.05); nd, not detected; ms, missing sample. [Colour figure can be viewed at wileyonlinelibrary.com]

In *G. biloba* seedlings, the expression levels of *GbGS1a*, *GbGS2* and *GbGS1b* (1–3) were quantified during the L/D cycle and when plants were placed into continuous darkness (Figure 4a). The absence of leaves in *G. biloba* seedlings germinated under continuous darkness did not allow us to quantify the level of *GS* gene expression in this organ (García‐Gutiérrez et al., 1998). Therefore, light/dark transition experiments were carried out using the leaves of 1‐year‐old *G. biloba* plants (Figure 4b). The level of *GbGS2* transcripts was very low both in the stems and the roots when the seedlings were grown under L/D or continuous darkness conditions. In contrast, the expression level of *GbGS2* was at least 20‐fold higher in leaves grown under L/D conditions. Although the level of *GbGS1a* transcripts was higher in the leaves than in the other organs, it was four times lower than that of *GbGS2*. The three genes encoding *GbGS1b* were expressed at a higher level in the stems and in the roots than in the leaves. Two significant correlations were found: between the expression levels of *GbGS1a* and *GbGS2* (0.9) and between the expression levels of *GbGS1b.1* and *GbGS1b.2* (0.89).

When fully expanded leaves were used, *GbGS1a* exhibited the highest level of expression compared with all the other *GS* genes during the L/D cycle. The pattern of *GbGS2* gene expression was similar to that of *GbGS1a*, although the transcript accumulation was three times lower. Transcripts for *GbGS1b.3* were not detected, irrespective of the light/dark regime (Figure 4b).

**Figure 5:**
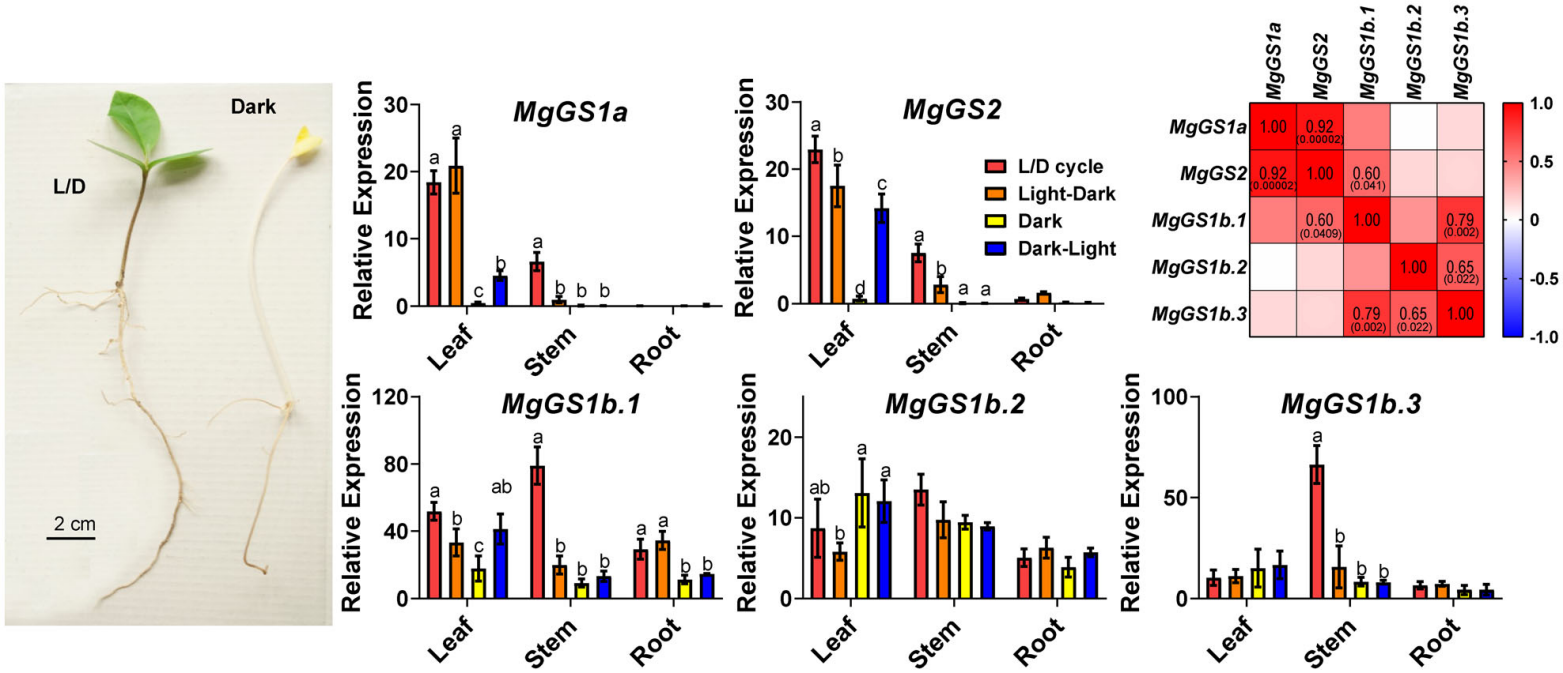
Glutamine synthetase (GS) gene expression in *Magnolia grandiflora* seedlings grown under different light regimes: L/D cycle (16‐h light/8‐h dark photoperiod, red bars); transition from light photoperiod to continuous darkness (orange bars); continuous darkness (yellow bars); and transition from complete darkness to light photoperiod (blue bars). Significant differences were determined using a two‐way analysis of variance (anova) that compares the mean for each condition with the mean of the other condition for the same organ. Letters above the columns indicate significant differences on a Tukey’s *post hoc* test (*P* < 0.05). Pearson’s correlation test was used to test for correlations between the expression levels of the different genes expressed in *M. grandiflora*. The significant *P* values (<0.05) are indicated in brackets. [Colour figure can be viewed at wileyonlinelibrary.com]

*Magnolia grandiflora* seedlings were also exposed to different light treatments to study the *GS* gene expression pattern in this species (Figure 5). The transcripts of *MgGS1a* and *MgGS2* were more abundant in the leaves than in the stems and roots, and their levels were similar for *MgGS1a* in the L/D cycle and light–dark treatments. A very low level of expression was obtained for *MgGS1a* when seedlings were placed under continuous darkness. Its level of expression was approximately fourfold lower than that of the L/D and light/dark treatments following a dark–light transition. *MgGS2* and *MgGS1a* exhibited a similar pattern of transcript accumulation, except that for *MgGS2* there was a significant decrease in the light–dark treatment and an increase during the transfer from dark to light. *MgGS1b.1* was the gene exhibiting the highest level of expression compared with all the other genes encoding GS. Its pattern of expression in the different organs was similar to that of *MgGS2*. However, the levels of *MgGS1b.1* transcripts were much higher in the stems, notably in L/D conditions, and in the roots. *MgGS1b.2* expression levels were similar in the three organs. No marked differences between the light and dark treatments were observed for this gene. *MgGS1b.3* transcript accumulation was similar irrespective of the organ and light/dark regimes, except in the stem, in which it was much higher during the L/D cycle. As shown in Figure 5, only four significant correlations were found between the expression level of the gene encoding GS in magnolia, where the highest correlation was between *MgGS1a* and *MgGS2* (0.92).

## DISCUSSION

In all plant species, each GS isoenzyme plays a key role, either in primary N assimilation or N recycling, as most of the N‐containing molecules required for growth and development are derived from glutamine, the product of the reaction catalyzed by this enzyme. Throughout evolution, such important metabolic functions are subject to high selective pressure, which made GS particularly suitable for plant phylogenetic analyses. However, our knowledge of the phylogeny of the GS isoenzymes gathered in the evolutionary group called GSII remains limited. The aim of the present study was thus to improve our knowledge on the classification and phylogeny of the Viridiplantae GSII. This study was performed using the corresponding gene sequences belonging to the main clades that are representative of plant evolution and covering a large portfolio of species, not limited to the model and crop angiosperms.

In the present investigation, the resulting phylogenetic analysis agreed with previous studies in which two main groups of plant GSII encoded by nuclear genes were identified, namely GSIIb (GLN2) and GSIIe (GLN1/GS) (Figures 1 and 2). An HGT event from eubacteria was previously proposed as the more parsimonious process for the emergence of the GLN2 group (Tateno, 1994; Ghoshroy et al., 2010). This hypothesis is further supported by the fact that we identified a signal peptide in all members of GLN2 that allows the targeting of the proteins to organelles such as plastids and mitochondria (Table S1). Interestingly, genes encoding GLN2 were not identified in vascular plant (tracheophyte) species. Therefore, GLN2 seemed to be lost, coinciding with the final adaptation of plants to land habitats. Such an adaptive mechanism notably included the development of vascular structures for assimilate transport and the presence of lignin involved in plant stature (Raven, 2018; Renault et al., 2019). In fact, the massive production of lignin, a metabolic feature of vascular plants, was enabled by a deregulation of phenylalanine biosynthesis that occurred at some point during the evolution of non‐vascular plants and tracheophytes (El‐Azaz et al., 2022). These developmental and regulatory processes could also be related to the selection of the *GLN1*/*GS* genes in the most ancient vascular plants. Thus, it was hypothesized that GLN1/GS isoenzymes are involved: (i) in the synthesis of the transport of glutamine and derived amino acids (Bernard and Habash, 2009); and (ii) in the production of monolignols used as precursors for lignin biosynthesis. As lignin represents one of the main sinks for the photosynthetic carbon assimilated by the plant, high levels of GS activity are thus required to assimilate the large quantities of ammonium released during the reaction catalyzed by the enzyme phenylalanine ammonia lyase (Pascual et al., 2016). The phylogenetic analyses performed in the present study suggest that the group represented by GLN1 isoenzymes can be considered the starting point for the evolution of the most recent genes encoding GS in plants.

The phylogeny of the ancient Embryophyta clades (Anthocerotophyta, Bryophyta and Marchantiophyta) suggests that the current GS subgroups in Spermatophyta clades were not established in non‐vascular land plants (Figures 1 and 2). Curiously, GLN2 was also found in these three clades, which could be the result of a stable situation related to the interaction of genotypic and environmental conditions during the expansion of this group of plants. However, a different clustering of GS in Lycopodiopsida and Polypodiopsida was observed between gene and protein phylogenetic trees, resulting in an unclear phylogenetic relationship (Figures 1 and 2). This finding suggests that during plant evolution there was an active adaptation process that resulted from changes in environmental conditions, such as an increase in the O_2_/CO_2_ ratio (Renault et al., 2019), and the loss of the *GLN2* gene. According to this hypothesis, several Lycopodiopsida and Polypodiopsida GS protein sequences contain a predicted transit peptide allowing its import into plastids (Table S1). Therefore, under an oxygen‐enriched atmosphere, the occurrence of plastidic GS seems to be beneficial for the plant, in turn leading to positive selection.

We also refined the classification of GS in seed plants (Spermatophyta), leading to the identification of three distinct clusters. One was the well‐known group of genes from angiosperms encoding plastidic GS (GS2) (for a review, see Hirel and Krapp, 2021). The two other clusters contained the genes encoding cytosolic GS (GS1). One of the clusters included all the GS1 isoenzymes classically found in angiosperms and the GS1b from gymnosperms (Cánovas et al., 2007; Bernard and Habash, 2009). The third group included the GS1a sequences from gymnosperms, including ginkgo (Cantón et al., 1993; Ávila‐Sáez et al., 2000), and different GS1 sequences from basal angiosperms and some Magnoliidae species. We thus propose to modify the nomenclature of *GS* genes from spermatophyte species into three subsets, namely *GS1a*, *GS1b* and *GS2*. Consequently, GSIIe can be used as a good phylogenetic marker in seed plants, as the presence or absence of the different *GS* gene groups is characteristic of the main taxa.

Surprisingly, searches for GS sequences in the NGS data from public databases allowed us to identify genes encoding GS2 in Cycadopsida species, contrary to previous findings (Miyazawa et al., 2018). Such a finding was experimentally confirmed by cloning a cDNA encoding GS2 from *Cycas revoluta* (MZ073670). The obtained sequence of the cloned *GS2* cDNA from *C. revoluta* validated the assembly of the *Cycas hainanensis* sequence using public NGS data (Figures S3 and S4). Consequently, this result demonstrated that there are more plant clades that possess GS2, which forms a new perspective on the evolution of GS2. In line with such a finding, recent phylogenomic studies showed that Cycadopsida and Ginkgoopsida formed a monophyletic group (Wu et al., 2013; Li et al., 2017; One Thousand Plant Transcriptomes Initiative, 2019). The presence of the genes encoding GS2 in both clades and its absence in the other gymnosperm clades (Coniferopsida and Gnetopsida) supports this taxonomic classification.

**Figure 6:**
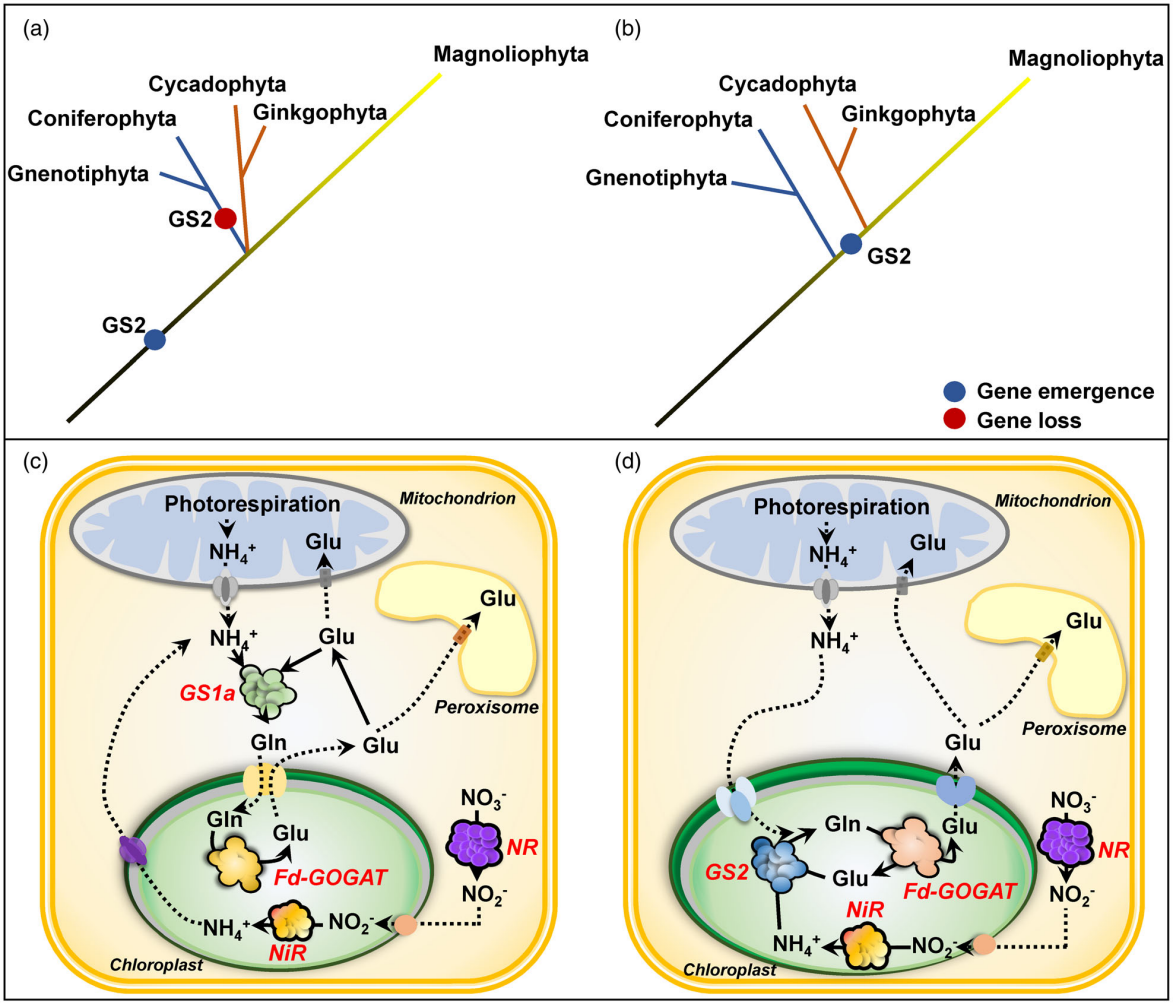
Schematic representation of *GS2* emergence hypotheses: (a) two‐event hypothesis; (b) single‐event hypothesis. (c) Simplified metabolic pathways in a photosynthetic cell in which ammonium assimilation is catalyzed by GS1a. (d) Metabolic pathway of a photosynthetic cell in which ammonium assimilation is catalyzed by GS2. GS, glutamine synthetase; Fd‐GOGAT, ferredoxin‐dependent glutamate synthase; NR, nitrate reductase; NiR, nitrite reductase. [Colour figure can be viewed at wileyonlinelibrary.com]

Based on our phylogenetic analysis, two hypotheses can be proposed concerning GS2 emergence and evolution.

i A two‐event evolutionary process, in which the gene encoding GS2 arose from a common ancestor of gymnosperms and angiosperms (Figure 6a). GS1 from Polypodiopsida, which is more closely related to GS2, could have been the origin of the plastid isoform following a specialization process that included the addition of a sequence that allowed the protein to be imported into the plastids. This hypothesis implies that there was a second genetic event consisting of the loss of GS2 in the common ancestor of Coniferopsida and Gnetopsida plants.
ii A single event during which GS2 sequences emerged from a common ancestor of Cycadopsida/Ginkgoopsida and angiosperm clades, leaving Coniferopsida and Gnetopsida without this gene (Figure 6b). Although gymnosperms are considered a monophyletic clade that is sister to angiosperms (One Thousand Plant Transcriptomes Initiative, 2019), the single‐event hypothesis for GS2 evolution is more parsimonious and suggests a revision of the phylogenetic relationships of the Cycadopsida/Ginkgoopsida clade with angiosperms.

The occurrence of a gene encoding GS1a has never been previously described in ginkgo, basal angiosperms and Magnoliidae species. The physiological function of GS1a was extensively studied in conifers because it compensates for the lack of GS2. Although GS1a is a cytosolic form of the enzyme, its light‐dependent expression level is also associated with chloroplast development, photorespiration, and N assimilation and recycling in photosynthetic tissues (Cánovas et al., 2007). One can hypothesize that, even though they are in different cellular compartments, GS1a and GS2 play redundant roles in photosynthetic cells, which could explain the disappearance of GS1a in the most recent angiosperm species. The light dependence and organ gene expression of *GS1a* and *GS2* in *P. pinaster* (Figure 3), *G. biloba* (Figure 4) and *M. grandiflora* (Figure 5) strengthened the previous hypothesis that GS1a might fulfill the function of GS2 (Cantón et al., 1999; Ávila et al., 2001; Gómez‐Maldonado et al., 2004). Interestingly, one of the two Cys residues involved in the redox modulation of GS2 activity (e.g. C306 in *Arabidopsis*) was conserved in all of the GS1a proteins (Cantón et al., 1993; Choi et al., 1999; Miyazawa et al., 2018). We also observed that this residue was conserved in all the GS1a and GS2 protein sequences analyzed in this study, and in sequences from Polypodiopsida (ancient Embryophyta clades), several Lycopodiopsida species and GLN1sequences (Figure S5). Remarkably, this Cys residue is absent in all GS1b sequences, suggesting a specific role of this residue in the function of GS2 and GS1a. In angiosperms, a second Cys residue is present in GS2 (e.g. C371 in *Arabidopsis*) and in several GLN1 proteins. Moreover, this second Cys residue is not present in ginkgo or Cycadopsida GS2, which suggests that it was acquired by angiosperms during plant evolution (Figure S5).

When the single‐event hypothesis is considered, the emergence of GS2 sequences following the loss of GS1a sequences in recent angiosperms indicates that they probably have redundant physiological functions. The Cycadopsida/Ginkgoopsida group emerged at least 270 Mya (Wu et al., 2013; Li et al., 2017; One Thousand Plant Transcriptomes Initiative, 2019) during the Permo‐Carboniferous period, when the atmospheric oxygen level rose from 21 to 35%. Such an elevation in oxygen level led to a drastic increase in the oxygenase activity of the photosynthetic enzyme Rubisco, leading to increased photorespiration (Berling and Bermer, 2000). The series of events that occurred during the Permo‐Carboniferous period could result in the appearance of a plastidic GS isoenzyme (GS2) through a positive selection process, allowing for a more efficient reassimilation of ammonium that is released during photorespiration. The mutation of GS2 in several species induced lethality under photorespiratory conditions (Blackwell et al., 1987; Wallsgrove et al., 1987; Pérez‐Delgado et al., 2015), which is not the case in *Arabidopsis*, as it can cope with the toxicity of ammonium released from the photorespiratory pathway (Ferreira et al., 2019; Hachiya et al., 2021). Conifers, which are C3 species possessing only cytosolic GS isoenzymes (Figure 6c), are also able to reassimilate the ammonium released during photorespiration. However, the increase in the level of atmospheric oxygen during the Permo‐Carboniferous period would imply an increase in nitrification rates, as oxygen is a substrate for nitrification (Ward, 2008), thus leading to an increase in nitrate availability in the rhizosphere. One can therefore hypothesize that such an increase in nitrate availability induced additional evolutionary pressure towards the selection of plastidic GS (GS2), which, in addition to photorespiratory ammonium reassimilation, is also responsible for the assimilation of ammonium in plastids derived from nitrate reduction (Figure 6d) (Hirel and Krapp, 2021). Consistent with this hypothesis, it is known that most conifers prefer or tolerate ammonium as an inorganic N source, which can be readily assimilated by cytosolic GS in the absence of GS2.

Concerning the process of GS2 selection, the most likely hypothesis is the duplication of genes encoding cytosolic GS, leading to functional specialization through changes such as those in the gene promoter and the addition of a sequence encoding a signal peptide used to import the protein into the chloroplasts (Biesiadka and Legocki, 1997; Ávila‐Sáez et al., 2000). Gene expression patterns, specific Cys residue conservation and nucleotide sequence‐based GS phylogeny suggest that *GS1a* could be at the origin of *GS2* (Figures 1 and 3, 4, 5).

Finally, we were able to conclude that the group represented by GS1b evolved in a different way than the groups represented by GS1a and GS2. In ginkgo and angiosperms, GS1b is generally represented by a small multigene family, with each member playing distinct roles either in N assimilation or N recycling, depending on the organ examined (Thomsen et al., 2014). In contrast, there is usually only one gene of GS1a or GS2, strongly suggesting that *GS1b* genes play non‐redundant roles compared with *GS1a* and *GS2* (Ghoshroy et al., 2010; Hirel and Krapp, 2021). Related to their different roles, the comparison between the GS1a and GS1b proteins in pine showed distinctive characteristics, such as a higher thermal stability of GS1b (de la Torre et al., 2002).

### Conclusions

The combined phylogenetic analysis and gene expression study presented in this work allowed us to improve our understanding of GS evolution in plants, notably in the Spermatophyta clade. In agreement with previous studies, two distinct groups of GS, GSIIb (GLN2) and GSIIe (GLN1 and GS), were clearly identified (Tateno, 1994; Ghoshroy et al., 2010).

An original finding of our study was that in seed plants, GS is represented by three types of genes (*GS1a*, *GS1b* and *GS2*), which allowed us to redefine the nomenclature of GS1 isoenzymes. In particular, the taxa possessing a gene encoding GS1a, originally represented by conifers (Cantón et al., 1993, 1999), are now expanded to ginkgo, basal angiosperms and some Magnoliidae species. In contrast, GS1a is not present in the most recent angiosperm species such as monocots and eudicots. Therefore, GS1a represents a new functional group as this cytosolic GS isoenzyme can compensate for the lack of GS2 (Ávila et al., 2001).

Unexpectedly, a gene encoding GS2 was identified in Cycadopsida species. This new finding supports the hypothesis that this clade, together with Ginkgoopsida species, represents a monophyletic group (Wu et al., 2013). Concerning the emergence and evolution of *GS2*, we provide strong lines of evidence that this gene arose in the most recent common ancestor of Cycadopsida, Ginkgoopsida and angiosperms. However, additional analyses including *GS* gene sequences from ferns will be required to confirm this hypothesis.

Beyond the numerous studies already performed in crops, the present investigation proposes new possibilities for a better understanding of the evolution of *GS* genes and their functions in terrestrial plants. Additional studies of GS evolution could help further decipher the impact of the different GS isoenzymes on N use efficiency to improve plant growth and productivity under a wide range of environmental conditions.

## EXPERIMENTAL PROCEDURES

### Glutamine synthetase gene sequences and phylogenetic analyses

Nucleotide sequences of the genes encoding plant glutamine synthetase type II (GSII) were obtained from different public databases or assembled from transcriptomic NGS data from the SRA database at the National Center for Biotechnology Information (NCBI, https://www.ncbi.nlm.nih.gov) and at the European Nucleotide Archive (ENA, https://www.ebi.ac.uk/ena) (Table S1). The tool employed for sequence search was BLAST (Altschul et al., 1990) using mainly the *tblastn* mode with the sequence of GS1b.1 from *Pinus taeda* as the query. For the assembly of sequences from NGS data, the raw files were uploaded to the web platform Galaxy (https://usegalaxy.org), which was used to make the transcriptome assemblies (Afgan et al., 2018). The raw reads were quality trimmed using trimmomatic (Bolger et al., 2014). The transcriptome assemblies were conducted with the trinity assembler (Grabherr et al., 2011) and the GS sequences were identified using BLAST, as described above. Database identifiers, names and species for the different GS sequences are presented in Table S1. All nucleotide and protein sequences used in the present work are available in Datasets S1 and S2, respectively. The subcellular localization prediction was determined with targetp (Almagro Armenteros et al., 2019) and localizer (Sperschneider et al., 2017).

For Bayesian phylogenetic analysis a data set of 169 nucleotide sequences encoding GSII from 45 different Viridiplantae species and *glnA* from *E. coli* were aligned with muscle (Edgar, 2004). Positions with gaps were deleted, and mrmodeltest 2.4 was used to find the best‐fitting model among the 24 models used to study molecular evolution (Nylander, 2004). The Akaike information criterion suggested the use of the model GTR + I + G. The Bayesian phylogenetic analysis was performed using mrbayes 3.2.7 (Huelsenbeck and Ronquist, 2001 ) with two simultaneous runs of 77 million generations for each run, with one cold and three heated chains for each run in which the temperature parameter was set to 0.1. Trees were sampled once every 10 000 generations. The average standard deviation of split frequencies at the end of each run was <0.01, and the first 25% of the trees were discarded as burn‐in samples. The consensus tree was visualized with the interactive tree of life (itol) web tool (Letunic and Bork, 2019). Detailed results of the Bayesian analysis are available in Dataset S3.

For maximum‐likelihood analysis, the data set was composed of 169 GS protein sequences obtained from the corresponding nucleotide sequences used for the Bayesian analyses. The alignment and phylogenetic analysis were conducted using mega 7 (Kumar et al., 2016). The sequences were aligned with muscle (Edgar, 2004). Maximum‐likelihood analyses were carried out using the complete deletion of gaps, the missing data, and the Jones–Taylor–Thornton (JTT) amino acid substitution model (Jones et al., 1992). Nearest‐neighbor interchange (NNI) was used for tree inference. The initial tree was constructed using the NJ/BioNJ method. The phylogeny test was performed using the bootstrap method with 1000 replications. Detailed results of the maximum‐likelihood analysis are available in Dataset S4.

### Plant material

*Ginkgo biloba* seeds were obtained from several botanic gardens: Botanische Gärten der Universität Bonn (Bonn, Germany), Botanischer Garten der Universität Bern (Bern, Switzerland), Plantentuin Universteit Gent (Ghent, Belgium) and Arboretum Wespelaar (Wespelaar, Belgium). Ginkgo seeds were stratified for 3 months in vermiculite at 4°C. *Pinus pinaster* seeds from Sierra Bermeja (Estepona, Spain) (ES20, ident. 11/12) were obtained from the Red de Centros Nacionales de Recursos Genéticos Forestales of the Spanish Ministerio para la Transición Ecológica y el Reto Demográfico with authorization number ESNC87. Pine seeds were imbibed for 72 h under continuous aeration with an air pump*. Magnolia grandiflora* seeds were obtained from the Parque de la Alameda garden in Málaga (Spain) and from private suppliers. Magnolia seeds were stratified in vermiculite at 4°C for 4 months. All seeds were growth on vermiculite in order to prevent any nutritional effect of the substrate.

Ginkgo, pine and magnolia seedlings were germinated and grown at 23°C with a 16‐h light/8‐h dark photoperiod, and watered once every 3 days with distillated water, or under continuous darkness, and watered once a week. For light/dark transition experiments, seedlings grown in complete darkness were transferred to a 16‐h light/8‐h dark photoperiod for 24 h and seedlings grown with a 16‐h light/8‐h dark photoperiod were transferred to complete darkness for 24 h. The leaves, stems and roots of the seedlings were harvested separately. For ginkgo seedlings grown in complete darkness the primary and secondary leaves did not develop, and thus only stems and roots were harvested. To study the impact of a light–dark transition on the expression of the different genes encoding GS in ginkgo leaves, one‐year‐old plants with fully developed leaves were used. These plants were first exposed to a 16‐h light /8‐h dark photoperiod, then to complete darkness for 24 h and then back to a 16‐h light/8‐h dark photoperiod. The different plant samples were immediately frozen in liquid N and stored at −80°C.

For the cloning of *Cycas revoluta GS2*, leaves from one‐year‐old plants grown under a 16‐h light/8‐h dark photoperiod were harvested. Leaf tissues were frozen immediately in liquid N and stored at −80°C until further use for RNA extraction.

### RNA extraction and reverse transcription quantitative polymerase chain reaction (RT‐qPCR)

Ginkgo and magnolia RNAs were extracted using the Plant/Fungi Total RNA Purification Kit (Norgen Biotek Corp., https://norgenbiotek.com) according to the manufacturer’s instruction. Pine and *Cycas* RNAs were extracted as described by Canales et al. (2012). For the cDNA synthesis, 500 ng of total RNA was used and retrotranscribed using the iScrpt™ Reverse Transcription Supermix (Bio‐Rad, https://www.bio‐rad.com). qPCR was carried out using 10 ng of cDNA and the SsoFast™ EvaGreen^®^ Supermix (Bio‐Rad). The reaction was carried out in a thermal cycler CFX384™ Touch Real‐Time PCR (Bio‐Rad). The analyses were carried out as described by Cañas et al. (2014) using the MAK3 model in the r package qpcr (Ritz and Spiess, 2008). For the RT‐qPCR analysis, three technical replicates of each sample and three biological replicates were performed. The results for maritime pine were normalized using a *saposin‐like aspartyl protease* (unigene1135) as a reference gene (Granados et al., 2016). Several references genes used to study gene expression in maritime pine (Granados et al., 2016) were tested in gingko and magnolia. In this species, the orthologs of maritime pine *saposin‐like aspartyl protease* (unigene1135), *myosin heavy chain‐related* (unigene13291) and of an RNA binding protein (unigene27526) were selected and used to normalize the expression of the genes encoding GS. In magnolia, the ortholog of an *RNA binding protein* (unigene27526) from maritime pine and *Actin‐7* from magnolia (Lovisetto et al., 2015) were selected and used as reference genes. The different primers used for the RT‐qPCR experiments are presented in Table S2. The magnolia and ginkgo sequences used to design the primers are listed in Table S3.

A *GS2* cDNA from *C. revoluta* was cloned using a PCR product. iProof™ HF Master Mix (Bio‐Rad) was used to perform the PCR reaction. Primer sequences were obtained from *C. hainanensis* and presented in Table S2. After the initial denaturation step at 98°C for 1 min, the PCR was conducted for 35 cycles with the following conditions: 10 s at 98°C; 20 s at 60°C and 1 min at 72°C, with a final extension step at 72°C for 5 min. The resulting PCR product was cloned into the pJET1.2 cloning vector (ThermoFisher Scientific, https://www.thermofisher.com). The sequence of *GS2* from *C. revoluta* was submitted to GenBank (MZ073670).

### Statistics

Statistical analyses were performed using prism 8 (Graphpad, https://www.graphpad.com). Data obtained from gene expression quantification were analyzed using a multiple comparison two‐way analysis of variance (ANOVA) test. Differences between organs were not analyzed statistically. Tukey’s *post hoc* test was used for the statistical analysis of the gene expression data. For *G. biloba* and *M. grandiflora* seedlings a Pearson correlation test was also used to evaluate the relationships between the expressions of the different *GS* genes. Differences and correlations were considered to be significant when the *P* value was <0.05.

## AUTHOR CONTRIBUTIONS

JMVM and FO performed the experiments. FRC and RAC performed the phylogenetic analysis. JMVM, BH, FRC and RAC wrote the article. JMVM, FO and RAC designed the figures. FMC and CA made additional contributions and edited the article. RAC, CA and FMC were responsible of funding acquisition. FRC and RAC planned and designed the research.

## CONFLICT OF INTEREST

The authors declare that they have no conflicts of interest associated with this work.

### OPEN RESEARCH BADGES

This article has earned an Open Data badge for making publicly available the digitally‐shareable data necessary to reproduce the reported results. The data are available at Cycas revoluta GS2 sequence NCBI's GenBank: https://www.ncbi.nlm.nih.gov/nuccore/MZ073670.1/.

## Supporting information


**Figure S1.** Phylogenetic tree obtained following Bayesian analysis of the GS nucleotide sequences in which branch lengths are maintained.

**Figure S2.** Phylogenetic tree obtained following a Bayesian analysis of the GS protein sequences in which branch lengths are maintained.

**Figure S3.** Multiple sequence alignment of the GS2 protein sequences from *Cycas revoluta* (CrGS2) and *Cycas hainanensis* (ChaGS2).

**Figure S4.** Multiple sequence alignment of the complete coding DNA sequences (CDSs) of the genes encoding GS2 from *Cycas revoluta* (*CrGS2*) and *Cycas hainanensis* (*ChaGS2*).

**Figure S5.** Multiple sequence alignment of the protein regions around the Cys residues involved in the redox modulation of GS2 activity with C306 and C371 positions in Arabidopsis GS2.

**Table S1.** Sequences names, accession numbers, species taxonomy data and putative subcellular localization of the different encoded GSs.

**Table S2.** List of primers used for RT‐qPCR experiments on *CrGS2* cloning.

**Table S3.** Magnolia and ginkgo GS gene sequences used to design the primers used for RT‐qPCR experiments.

**Data S1.** GS nucleotide sequences used to perform the phylogenetic analyses.

**Data S2.** GS protein sequences used to perform the phylogenetic analyses.

**Data S3.** Detailed results of the Bayesian analysis using the GS gene nucleotide sequences.

**Data S4.** Detailed results of the maximum‐likelihood analysis using the GS protein sequences.

## Data Availability

The data that support the findings of this study are available from different databases, the supporting information and from the corresponding author, upon reasonable request.
