# Supplementary material for: A revised view on the evolution of glutamine synthetase isoenzymes in plants
Source: Plant J. 2022 Mar 9;110(4):946–60. doi: 10.1111/tpj.15712 (PMC9310647; doi:10.1111/tpj.15712)
Supplement: Supplementary file 1 — Figure S1. Phylogenetic tree obtained following Bayesian analysis of the GS nucleotide sequences in which branch lengths are maintained. [file TPJ-110-946-s004.pdf]

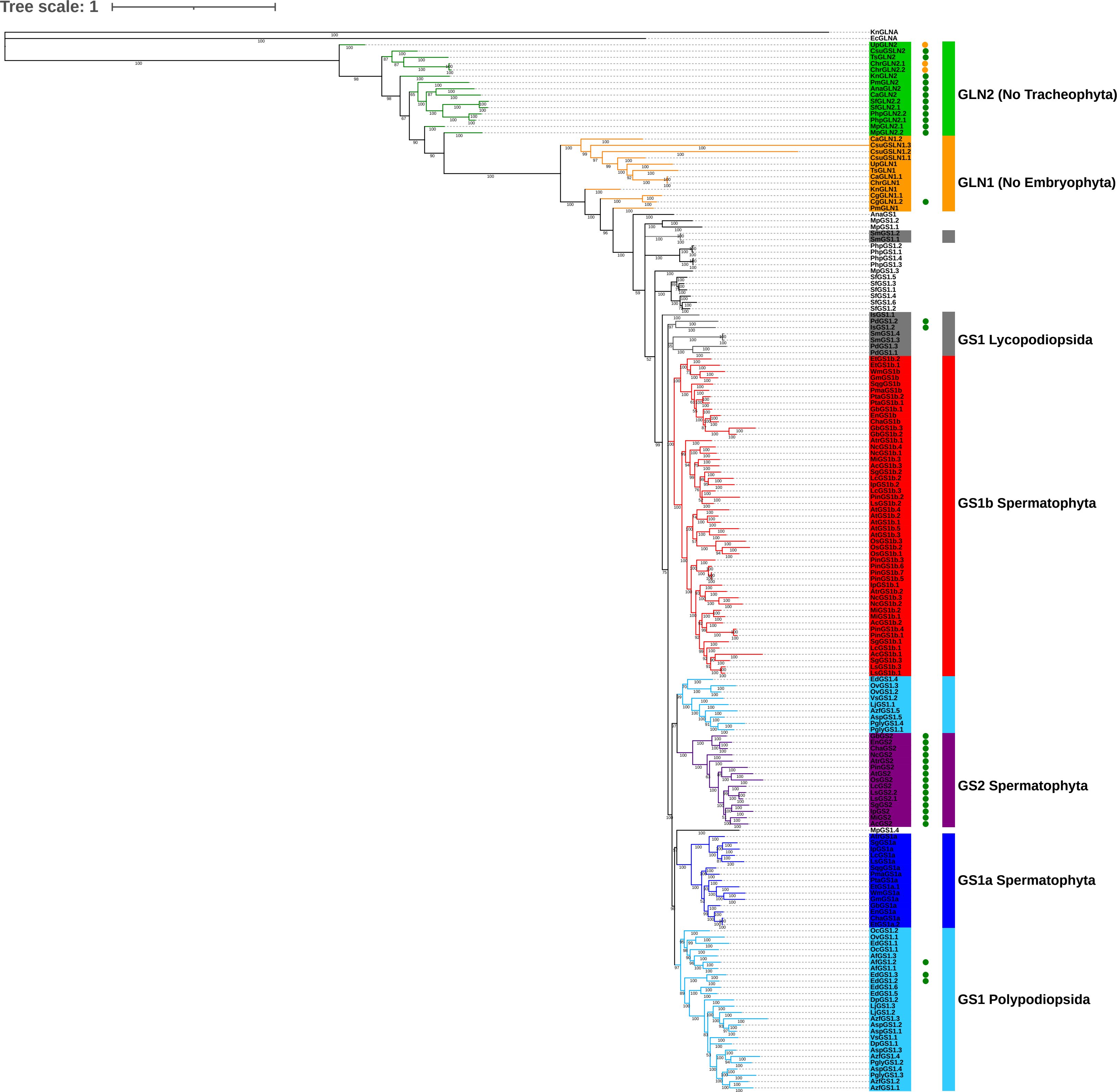
Tree scale: 1
KnGLNA
EcGLNA
UpGLN2
CsuGSLN2
TsGLN2
ChrGLN2.1
ChrGLN2.2
KnGLN2
PmGLN2
AnaGLN2
CaGLN2
SfGLN2.2
SfGLN2.1
PhpGLN2.2
PhpGLN2.1
MpGLN2.1
MpGLN2.2
CaGLN1.2
CsuGSLN1.3
CsuGSLN1.2
CsuGSLN1.1
UpGLN1
TsGLN1
CaGLN1.1
ChrGLN1
KnGLN1
CgGLN1.1
CgGLN1.2
PmGLN1
AnaGS1
MpGS1.2
MpGS1.1
SmGS1.2
SmGS1.1
PhpGS1.2
PhpGS1.1
PhpGS1.4
PhpGS1.3
MpGS1.3
SfGS1.5
SfGS1.3
SfGS1.1
SfGS1.4
SfGS1.6
SfGS1.2
IsGS1.1
PdGS1.2
IsGS1.2
SmGS1.4
SmGS1.3
PdGS1.3
PdGS1.1
EtGS1b.2
EtGS1b.1
WmGS1b
GmGS1b
SqgGS1b
PmaGS1b
PtaGS1b.2
PtaGS1b.1
GbGS1b.1
EnGS1b
ChaGS1b
GbGS1b.3
GbGS1b.2
AtrGS1b.1
NcGS1b.4
NcGS1b.1
MiGS1b.3
AcGS1b.3
SgGS1b.2
LcGS1b.2
IpGS1b.2
LcGS1b.3
PinGS1b.2
LsGS1b.2
AtGS1b.4
AtGS1b.2
AtGS1b.1
AtGS1b.5
AtGS1b.3
OsGS1b.3
OsGS1b.2
OsGS1b.1
PinGS1b.3
PinGS1b.6
PinGS1b.7
PinGS1b.5
IpGS1b.1
AtrGS1b.2
NcGS1b.3
NcGS1b.2
MiGS1b.2
MiGS1b.1
AcGS1b.2
PinGS1b.4
PinGS1b.1
SgGS1b.1
LcGS1b.1
AcGS1b.1
SgGS1b.3
LsGS1b.3
LsGS1b.1
EdGS1.4
OvGS1.3
OvGS1.2
VsGS1.2
LjGS1.1
AzfGS1.5
AspGS1.5
PglyGS1.4
PglyGS1.1
GbGS2
EnGS2
ChaGS2
NcGS2
AtrGS2
PinGS2
AtGS2
OsGS2
LcGS2
LsGS2.2
LsGS2.1
SgGS2
IpGS2
MiGS2
AcGS2
MpGS1.4
AtrGS1a
SgGS1a
IpGS1a
LcGS1a
LsGS1a
SqgGS1a
PmaGS1a
PtaGS1a
EtGS1a.1
WmGS1a
GmGS1a
GbGS1a
EnGS1a
ChaGS1a
OcGS1.2
OvGS1.1
EdGS1.1
OcGS1.1
AfGS1.3
AfGS1.2
AfGS1.1
EdGS1.3
EdGS1.2
EdGS1.6
EdGS1.5
DpGS1.2
LjGS1.3
LjGS1.2
AzfGS1.3
AspGS1.2
AspGS1.1
VsGS1.1
DpGS1.1
AspGS1.3
AzfGS1.4
PglyGS1.2
AspGS1.4
PglyGS1.3
AzfGS1.2
AzfGS1.1
GLN2 (No Tracheophyta)
GLN1 (No Embryophyta)
GS1 Lycopodiopsida
GS1b Spermatophyta
GS2 Spermatophyta
GS1a Spermatophyta
GS1 Polypodiopsida
