# Supplementary figures and images for: A revised view on the evolution of glutamine synthetase isoenzymes in plants
Source: Plant J. 2022 Mar 9;110(4):946–60. doi: 10.1111/tpj.15712 (PMC9310647; doi:10.1111/tpj.15712)

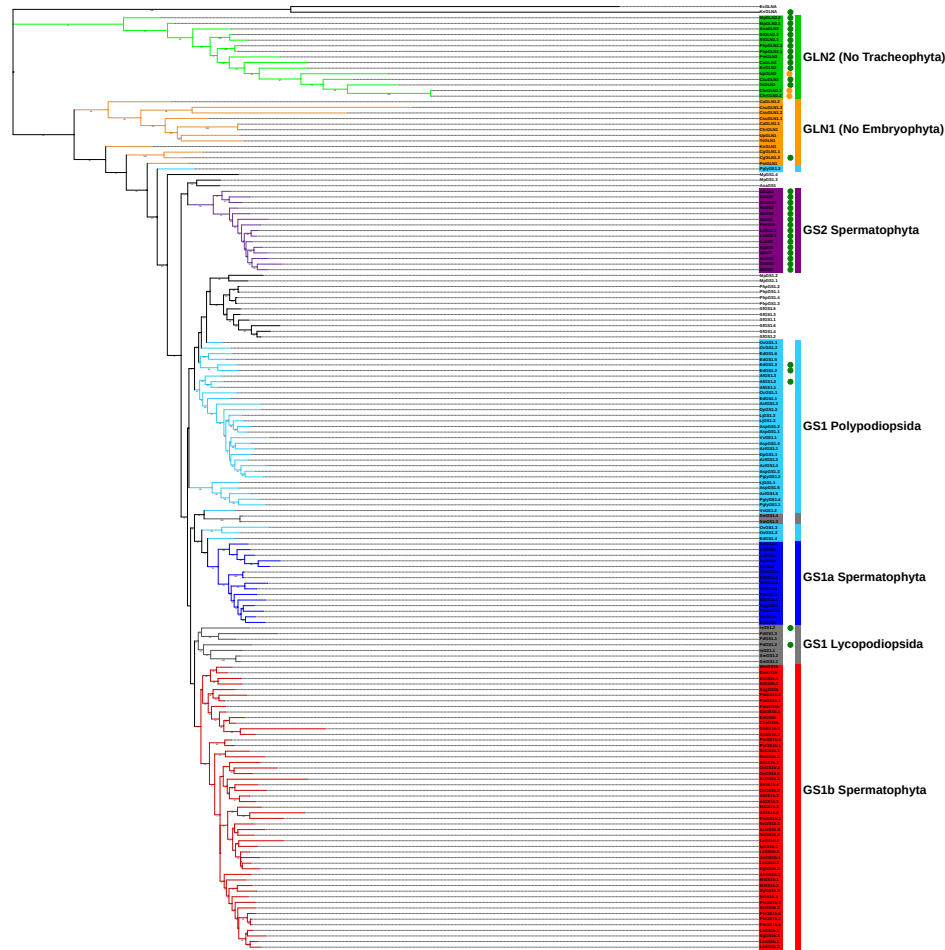

Supplement: Supplementary file 2 — Figure S2. Phylogenetic tree obtained following a Bayesian analysis of the GS protein sequences in which branch lengths are maintained. [file TPJ-110-946-s005.pdf]
