# Supplementary material for: A revised view on the evolution of glutamine synthetase isoenzymes in plants
Source: Plant J. 2022 Mar 9;110(4):946–60. doi: 10.1111/tpj.15712 (PMC9310647; doi:10.1111/tpj.15712)
Supplement: Supplementary file 3 — Figure S3. Multiple sequence alignment of the GS2 protein sequences from Cycas revoluta (CrGS2) and Cycas hainanensis (ChaGS2). [file TPJ-110-946-s007.pdf]

```
           1                                                                                                  100
CrGS2      MSQALVPSLQ WRILPGGVPM TATKMSNCLL PSRGVGLKSA PFNGRLSRSK RSTLHGRNYV SSVRADSVAW NPTENGSASR LIDLLNLDLS PFTDKVIAEY
ChaGS2     MSQALVPSLQ WRILPGGVPM TATKMSNGLL PSRGVGLKSA PFNGRLSRSK RSTLHGRNYV NSVRADSVAW NPTENGSASR LIDLLNLDLS PFTDKVIAEY
Consensus  MSQALVPSLQ WRILPGGVPM TATKMSNcLL PSRGVGLKSA PFNGRLSRSK RSTLHGRNYV nSVRADSVAW NPTENGSASR LIDLLNLDLS PFTDKVIAEY

           101                                                                                                200
CrGS2      LWIGGSGLDI RSKARTVSGP IDNPAKLPKW NYDGSSTGQA PGEDSEVILY PQAIFKDPFR GGNNILVICD SYKPNGEPIP TNKRANAAKI FSQKKVIDEE
ChaGS2     LWIGGSGLDI RSKARTVSGP IDNPAKLPKW NYDGSSTGQA PGEDSEVILY PQAIFKDPFR GGNNILVICD CYKPNGEPIP TNKRANAAKI FSQKKVIDEE
Consensus  LWIGGSGLDI RSKARTVSGP IDNPAKLPKW NYDGSSTGQA PGEDSEVILY PQAIFKDPFR GGNNILVICD cYKPNGEPIP TNKRANAAKI FSQKKVIDEE

           201                                                                                                300
CrGS2      PWYGIEQEYT LLQKNVKWPL GWPIGGYPGP QGPYYCGTGV DKAYGRVIAD AHYKACVYAG IKVSGINSEV MPGQWEYQVG PSVGIASGDH LWCSRYILER
ChaGS2     PWYGIEQEYT LLQKNVKWPL GWPIGGYPGP QGPYYCGTGV DKAYGRVIAD AHYKACVYAG IKVSGINSEV MPGQWEYQVG PSVGIASGDH LWCSRYILER
Consensus  PWYGIEQEYT LLQKNVKWPL GWPIGGYPGP QGPYYCGTGV DKAYGRVIAD AHYKACVYAG IKVSGINSEV MPGQWEYQVG PSVGIASGDH LWCSRYILER

           301                                                                                                400
CrGS2      ITEMAGVVLS LDPKPIEGDW NGAGCHTNYS TKSMREDGGY EVIKKAILNL GLRHKEHISA YGEGNERRLT GHHETANINA FSWGVANRGA SIRVGRETEK
ChaGS2     ITEMAGVVLS LDPKPIDGDW NGAGCHTNYS TKSMREDGGY EVIKKAILNL GLRHKEHISA YGEGNERRLT GHHETANINA FSWGVANRGA SIRVGRETEK
Consensus  ITEMAGVVLS LDPKPI#GDW NGAGCHTNYS TKSMREDGGY EVIKKAILNL GLRHKEHISA YGEGNERRLT GHHETANINA FSWGVANRGA SIRVGRETEK

           401                                          449
CrGS2      QGKGYLEDRR PASNMDPYVV TSMLAETTIL WEPAPEAGTH AAKELQLQI
ChaGS2     QGKGYLEDRR PASNMDPYVV TSMLAETTIL WEPAPEAGTH AAKELQLQI
Consensus  QGKGYLEDRR PASNMDPYVV TSMLAETTIL WEPAPEAGTH AAKELQLQI
```
