# Supplementary material for: A revised view on the evolution of glutamine synthetase isoenzymes in plants
Source: Plant J. 2022 Mar 9;110(4):946–60. doi: 10.1111/tpj.15712 (PMC9310647; doi:10.1111/tpj.15712)
Supplement: Supplementary file 5 — Figure S5. Multiple sequence alignment of the protein regions around the Cys residues involved in the redox modulation of GS2 activity with C306 and C371 positions in Arabidopsis GS2. [file TPJ-110-946-s006.pdf]

```
                        ..500   ▼   511.. ..583   ▼   594..
  1 EcGLNA           ...NGSGMHCHMSLS... ...SARNRSASIRIP...
  2 ChrGLN1          ...NGSG--GHTNYS... ...GVANRGCSIRVG...
  3 ChrGLN2.2        ...NGTG--AHTNFS... ...GVADRGSSIRIP...
  4 ChrGLN2.1        ...NGTG--AHTNFS... ...GVADRGSSIRIP...
  5 CsuGLN1.1        ...NGAG--GHTNFS... ...GVANRGASIRVG...
  6 CsuGLN2          ...NGTG--AHTNFS... ...GVADRGSSIRIP...
  7 CsuGLN1.2        ...AGTG--GHTNYS... ...GVGDRGASVRVG...
  8 CsuGLN1.3        ...SGNG--AAVKFS... ...GMENRNASIRIP...
  9 TsGLN2           ...NGTG--AHTNYS... ...GFADRGASIRIP...
 10 TsGLN1           ...NGAG--GHTNYS... ...GVANRGCSIRVG...
 11 UpGLN1           ...NGAG--GHTNYS... ...GVANRGCSIRIG...
 12 UpGLN2           ...NGAG--AHTNYS... ...GVADRGSSIRIP...
 13 CaGLN1.2         ...NGAG--CHTNYS... ...GVADRGKSIRVG...
 14 CaGLN2           ...NGTG--GHTNYS... ...GVADRGASIRIP...
 15 CaGLN1.1         ...NGSG--GHTNYS... ...GVANRGCSIRVG...
 16 KnGLN2           ...NGTG--AHTNYS... ...GVSDRGASIRIP...
 17 KnGLN1           ...NGAG--CHTNYS... ...GVANRGASIRVG...
 18 KnGLNA           ...AGSS--CHVHMS... ...SKDNRTAPFRI-...
 19 CgGLN1.2         ...NGAG--CHTNYS... ...GVANRGASIRVG...
 20 CgGLN1.1         ...NGAG--CHTNYS... ...GVANRGASIRVG...
 21 PmGLN1           ...NGAG--AHTNYS... ...GVANRGCSIRVG...
 22 PmGLN2           ...NGTG--AHTNYS... ...GVADRGASIRIP...
 23 AnaGS1           ...NGAG--CHTNYS... ...GVANRGASIRVG...
 24 AnaGLN2          ...NGAG--AHTNYS... ...GVADRGASIRIP...
 25 MpGS1.1          ...NGAG--CHTNFS... ...GVANRGSSIRVG...
 26 MpGS1.2          ...NGAG--CHTNFS... ...GVANRGASIRVG...
 27 MpGLN2.1         ...NGAG--AHTNYS... ...GVADRGASIRIP...
 28 MpGLN2.2         ...NGAG--AHTNFS... ...GVADRGASVRIP...
 29 MpGS1.3          ...NGAG--CHTNYS... ...GVANRGASVRVG...
 30 MpGS1.4          ...NGAG--CHTNYS... ...GVADRGASIRVG...
 31 SfGLN2.1         ...NGAG--AHTNYS... ...GVADRGASIRIP...
 32 SfGLN2.2         ...NGAG--AHTNYS... ...GVADRGASIRIP...
 33 SfGS1.1          ...NGAG--CHTNYS... ...GVANRGASVRVG...
 34 SfGS1.2          ...NGAG--CHTNYS... ...GVANRGASIRVG...
 35 SfGS1.3          ...NGAG--CHTNYS... ...GVANRGASIRVG...
 36 SfGS1.4          ...NGAG--CHTNYS... ...GVANRGASIRVG...
 37 SfGS1.5          ...NGAG--CHTNYS... ...GVANRGASIRVG...
 38 SfGS1.6          ...NGAG--CHANYS... ...GVGNRGVSIRVG...
 39 PhpGS1.1         ...NGAG--CHTNYS... ...GVANRGASVRVG...
 40 PhpGS1.2         ...NGAG--CHTNYS... ...GVANRGASVRVG...
 41 PhpGS1.3         ...NGAG--CHTNYS... ...GVANRGASVRVG...
 42 PhpGS1.4         ...NGAG--CHTNYS... ...GVANRGASVRVG...
 43 PhpGLN2.1        ...NGAG--AHTNYS... ...GVADRGASIRIP...
 44 PhpGLN2.2        ...NGAG--AHTNYS... ...GVADRGASIRIP...
 45 SmGS1.1          ...NGAG--CHANYS... ...GVANRGASVRVG...
 46 SmGS1.2          ...NGAG--CHTNYS... ...GVANRGASVRVG...
 47 SmGS1.3          ...NGAG--AHTNYS... ...GVANRGASVRVG...
 48 SmGS1.4          ...NGAG--AHTNYS... ...GVANRGASVRVG...
 49 IsGS1.2          ...NGAG--AHTNYS... ...GVANRGASIRVG...
 50 IsGS1.1          ...NGAG--AHTNYS... ...GVANRGASVRVG...
 51 PdGS1.1          ...NGAG--AHTNYS... ...GVANRGASIRVG...
 52 PdGS1.2          ...NGAG--AHTNYS... ...GVANRGASIRVG...
 53 PdGS1.3          ...NGAG--AHVNYS... ...GAGTRSTSVRVS...
 54 EdGS1.1          ...NGAG--CHTNYS... ...GVANRGASIRVG...
 55 EdGS1.5          ...NGAG--CHTNYS... ...GVANRGASVRVG...
 56 EdGS1.6          ...NGAG--CHTNYS... ...GVANRGASIRVG...
 57 EdGS1.2          ...NGAG--CHTNYS... ...GVANRGSSIRVG...
 58 EdGS1.3          ...NGAG--CHTNFS... ...GVANRGSSIRVG...
 59 EdGS1.4          ...NGAG--CHTNYS... ...GVANRGASVRVG...
 60 AfGS1.1          ...NGAG--CHTNYS... ...GVANRGASIRVG...
 61 AfGS1.3          ...NGAG--CHTNYS... ...GVANRGASIRVG...
 62 AfGS1.2          ...NGAG--CHTNYS... ...GVANRGASVRVG...
 63 OvGS1.1          ...NGAG--CHTNYS... ...GVANRGASIRVG...
 64 OvGS1.2          ...NGAG--CHANYS... ...GVANRGASVRVG...
 65 OvGS1.3          ...NGGG--CHTNYS... ...GVANRGTSIRIG...
 66 PglyGS1.1        ...NGAG--CHTNFS... ...GVADRGASIRVG...
 67 PglyGS1.2        ...NGAG--CHTNYS... ...GVANRGASIRVG...
 68 PglyGS1.3        ...NGAG--CHTNYS... ...GVANRGASIRVG...
 69 PglyGS1.4        ...NGAG--CHTNFS... ...GVANRGASVRVG...
 70 DpGS1.1          ...NGAG--CHTNYS... ...GVANRGASIRVG...
 71 DpGS1.2          ...NGAG--CHTNYS... ...GVANRGASIRVG...
 72 VsGS1.1          ...NGAG--CHTNYS... ...GVANRGASVRVG...
 73 VsGS1.2          ...NGAG--CHTNYS... ...GVANRGASVRVG...
 74 OcGS1.1          ...NGAG--CHTNYS... ...GVANRGASIRVG...
 75 OcGS1.2          ...NGAG--CHTNYS... ...GVANRGASIRVG...
 76 AzfGS1.1         ...NGAG--CHTNYS... ...GVANRGASIRVG...
 77 AzfGS1.2         ...NGAG--CHTNYS... ...GVANRGASIRVG...
 78 AzfGS1.3         ...NGAG--CHTNYS... ...GVANRGVSVRVG...
 79 AzfGS1.4         ...NGAG--CHTNYS... ...GVANRGASIRVG...
 80 AzfGS1.5         ...NGAG--CHTNFS... ...GVANRGASVRVG...
 81 LjGS1.1          ...NGAG--CHTNYS... ...GVANRGASVRVG...
 82 LjGS1.2          ...NGAG--CHTNYS... ...GVANRGASIRVG...
 83 LjGS1.3          ...NGAG--CHTNYS... ...GVANRGASIRVG...
 84 AspGS1.1         ...NGAG--CHTNYS... ...GVANRGASIRVG...
 85 AspGS1.2         ...NGAG--CHTNYS... ...GVANRGASIRVG...
 86 AspGS1.3         ...NGAG--CHTNYS... ...GVANRGASIRVG...
 87 AspGS1.4         ...NGAG--CHTNYS... ...GVADRGASIRVG...
 88 AspGS1.5         ...NGAG--CHTNYS... ...GVANRGASVRVG...
 89 GmGS1b           ...NGAG--AHTNYS... ...GVANRGASVRVG...
 90 GmGS1a           ...NGAG--CHSNYS... ...GVANRGASVRVG...
 91 WmGS1b           ...NGAG--AHTNYS... ...GVANRGASVRVG...
 92 WmGS1a           ...NGAG--CHANYS... ...GVANRGASVRVG...
 93 EtGS1b.2         ...NGAG--AHTNYS... ...GVANRGASVRVG...
 94 EtGS1b.1         ...NGAG--AHTNYS... ...GVANRGASVRVG...
 95 EtGS1a.1         ...NGAG--CHTNYS... ...GVANRGASVRVG...
 96 EtGS1a.2         ...NGAG--CHTNYS... ...GVANRGASVRVG...
 97 PmaGS1b          ...NGAG--AHTNYS... ...GVANRGASIRVG...
 98 PmaGS1a          ...NGAG--CHTNYS... ...GVANRGASVRVG...
 99 SqgGS1b          ...NGAG--AHTNYS... ...GVANRGASIRVG...
100 SqgGS1a          ...NGAG--CHTNYS... ...GVANRGASIRVG...
101 PtaGS1a          ...NGAG--CHTNYS... ...GVANRGASVRVG...
102 PtaGS1b.1        ...NGAG--AHTNYS... ...GVANRGASIRVG...
103 PtaGS1b.2        ...NGAG--AHTNYS... ...GVANRGASVRIG...
104 ChaGS1a          ...NGAG--CHTNYS... ...GVANRGASVRVG...
105 ChaGS1b          ...NGAG--AHANYS... ...GVANRGASIRVG...
106 ChaGS2           ...NGAG--CHTNYS... ...GVANRGASIRVG...
107 EnGS1a           ...NGAG--CHTNYS... ...GVANRGASIRVG...
108 EnGS2            ...NGAG--CHTNYS... ...GVANRGASIRVG...
109 EnGS1b           ...NGAG--AHANYS... ...GVANRGASVRVG...
110 GbGS1a           ...NGAG--CHTNYS... ...GVANRGASVRVG...
111 GbGS1b.1         ...NGAG--AHTNYS... ...GVANRGASIRVG...
112 GbGS1b.2         ...NGAG--AHTNYN... ...GVADRGASIRVG...
113 GbGS1b.3         ...KGGR--AHTNYS... ...GVAKREASIRAG...
114 GbGS2            ...NGAG--CHTNYS... ...GVANRGASIRVG...
115 AtrGS1a          ...NGAG--CHTNYS... ...GVANRGASVRVG...
116 AtrGS1b.1        ...NGAG--AHTNYS... ...GVANRGASVRVG...
117 AtrGS1b.2        ...NGAG--CHSNYS... ...GVANRGASIRVG...
118 AtrGS2           ...NGAG--CHTNFS... ...GVANRGCSIRVG...
119 NcGS1b.1         ...NGAG--AHTNYS... ...GVANRGASVRIG...
120 NcGS1b.2         ...NGAG--AHTNYS... ...GVANRGASIRVG...
121 NcGS1b.3         ...NGAG--AHTNYS... ...GVANRGASIRVG...
122 NcGS1b.4         ...NGAG--AHTNYS... ...GVANRGASIRVG...
123 NcGS2            ...NGAG--CHTNYS... ...GVANRGCSIRVG...
124 IpGS1b.2         ...NGAG--AHTNYS... ...GVANRGASIRVG...
125 IpGS2            ...NGAG--CHTNYS... ...GVANRGCSIRVG...
126 IpGS1b.1         ...NGAG--AHTNYS... ...GVANRGASIRVG...
127 IpGS1a           ...NGAG--CHTNYS... ...GVANRGASIRVG...
128 SgGS1b.1         ...NGAG--AHTNYS... ...GVANRGASIRVG...
129 SgGS1b.2         ...NGAG--AHTNYS... ...GVANRGASIRVG...
130 SgGS1a           ...NGAG--CHTNYS... ...GVANRGASIRVG...
131 SgGS2            ...NGAG--CHTNYS... ...GVANRGCSIRVG...
132 SgGS1b.3         ...NGAG--AHTNYS... ...GVANRGASIRVG...
133 LsGS1a           ...NGAG--CHTNYS... ...GVANRGASIRVG...
134 LsGS1b.1         ...NGAG--AHTNYS... ...GVANRGASIRVG...
135 LsGS1b.2         ...NGAG--AHTNYS... ...GVANRGASIRVG...
136 LsGS1b.3         ...NGAG--AHTNYS... ...GVANRGASIRVG...
137 LsGS2.1          ...NGAG--CHTNYS... ...GVANRGCSIRVG...
138 LsGS2.2          ...NGAG--CHTNYS... ...GVANRGCSIRVG...
139 PinGS1b.1        ...NGAG--AHTNYS... ...GVANRGASIRVG...
140 PinGS1b.2        ...NGAG--AHTNYS... ...GVANRGASVRIG...
141 PinGS1b.3        ...NGAG--AHTNYS... ...GVANRGASIRVG...
142 PinGS2           ...NGAG--CHTNYS... ...GVANRGCSIRVG...
143 PinGS1b.4        ...NGAG--AHTNYS... ...GVANRGASIRVG...
144 PinGS1b.5        ...NGAG--AHTNYS... ...GVANRGASIRVG...
145 PinGS1b.6        ...NGAG--AHTNYS... ...GVANRGASVRVS...
146 PinGS1b.7        ...NGAG--AHTNYS... ...GVANRGASIRVG...
147 LcGS1a           ...NGAG--CHTNYS... ...GVANRGASIRVG...
148 LcGS1b.1         ...NGAG--AHTNYS... ...GVANRGASIRVG...
149 LcGS1b.2         ...NGAG--AHTNYS... ...GIRNRAASIRVG...
150 LcGS1b.3         ...NGAG--AHTNYS... ...GVANRGASIRVG...
151 LcGS2            ...NGAG--CHTNYS... ...GVANRGCSIRVG...
152 AcGS1b.1         ...NGTG--AHTNFS... ...GVGNRAASIRVG...
153 AcGS1b.2         ...NGAG--AHCNYS... ...GVANRGASVRVG...
154 AcGS1b.3         ...NGAG--AHTNYS... ...GVANRGASIRVG...
155 AcGS2            ...NGAG--CHTNYS... ...GVANRGCSIRVG...
156 MiGS1b.1         ...NGAG--AHTNYS... ...GVADRGASIRVG...
157 MiGS1b.2         ...NGAG--AHTNYS... ...GVANRGASIRVG...
158 MiGS1b.3         ...NGAG--AHTNYS... ...GVANRGASIRVG...
159 MiGS2            ...NGAG--CHTNYS... ...GVANRGCSIRVG...
160 OsGS1b.1         ...NGAG--AHTNYS... ...GVANRGASVRVG...
161 OsGS1b.2         ...NGAG--AHTNFS... ...GVANRGASIRVG...
162 OsGS1b.3         ...NGAG--AHTNYS... ...GVANRGASVRVG...
163 OsGS2            ...NGAG--CHTNYS... ...GVANRGCSIRVG...
164 AtGS1b.1         ...NGAG--AHCNYS... ...GVANRGASIRVG...
165 AtGS1b.2         ...NGAG--AHTNYS... ...GVANRGASIRVG...
166 AtGS1b.3         ...NGAG--AHCNYS... ...GVANRGASVRVG...
167 AtGS1b.4         ...NGAG--AHTNYS... ...GVANRGASIRVG...
168 AtGS1b.5         ...NGAA--AHTNFS... ...GVADRGASVRVG...
169 AtGS2            ...NGAG--CHTNYS... ...GVANRGCSIRVG...
```
